# Predicting Greenhouse Gas Emissions and Soil Carbon from Changing Pasture to an Energy Crop

**DOI:** 10.1371/journal.pone.0072019

**Published:** 2013-08-21

**Authors:** Benjamin D. Duval, Kristina J. Anderson-Teixeira, Sarah C. Davis, Cindy Keogh, Stephen P. Long, William J. Parton, Evan H. DeLucia

**Affiliations:** 1 Energy Biosciences Institute, University of Illinois at Urbana-Champaign, Urbana, Illinois, United States of America; 2 Global Change Solutions, Urbana, Illinois, United States of America; 3 Natural Resource Ecology Laboratory, Fort Collins, Colorado, United States of America; 4 Department of Plant Biology, University of Illinois at Urbana-Champaign, Urbana, Illinois, United States of America; University of Nottingham, United Kingdom

## Abstract

Bioenergy related land use change would likely alter biogeochemical cycles and global greenhouse gas budgets. Energy cane (*Saccharum officinarum* L.) is a sugarcane variety and an emerging biofuel feedstock for cellulosic bio-ethanol production. It has potential for high yields and can be grown on marginal land, which minimizes competition with grain and vegetable production. The DayCent biogeochemical model was parameterized to infer potential yields of energy cane and how changing land from grazed pasture to energy cane would affect greenhouse gas (CO_2_, CH_4_ and N_2_O) fluxes and soil C pools. The model was used to simulate energy cane production on two soil types in central Florida, nutrient poor Spodosols and organic Histosols. Energy cane was productive on both soil types (yielding 46–76 Mg dry mass⋅ha^−1^). Yields were maintained through three annual cropping cycles on Histosols but declined with each harvest on Spodosols. Overall, converting pasture to energy cane created a sink for GHGs on Spodosols and reduced the size of the GHG source on Histosols. This change was driven on both soil types by eliminating CH_4_ emissions from cattle and by the large increase in C uptake by greater biomass production in energy cane relative to pasture. However, the change from pasture to energy cane caused Histosols to lose 4493 g CO_2_ eq⋅m^−2^ over 15 years of energy cane production. Cultivation of energy cane on former pasture on Spodosol soils in the southeast US has the potential for high biomass yield and the mitigation of GHG emissions.

## Introduction

Land use has a pervasive influence on atmospheric greenhouse gas (GHG) concentrations and thereby on climate [1], [2], [3]. Carbon emissions from land use change, often to make way for agriculture, have contributed substantially to anthropogenic increases in the atmospheric CO_2_ concentration [2]. For example, C emissions from tropical deforestation have been estimated at 10.6±1.8 Pg CO_2_ per year between 1990 and 2007, equal to ∼40% of global fossil fuel emissions [3]. Likewise, it is estimated that 40–52 Pg CO_2_ have been released by plowing high-C native prairie soils [4]. Agricultural practices are important to global GHG budgets, with agroecosystems contributing ∼14% of global anthropogenic GHG emissions [1]. Agricultural practices can also reduce GHG emissions and enhance soil carbon, and have the potential to mitigate climate change [4], [5].

Land use and land management changes associated with the emerging bioenergy industry are likely to have substantial impacts on global GHG budgets [6], [7], [8]. A change from fossil fuels to an energy economy more reliant on plant-derived biofuels has the potential to reduce GHG emissions [9]. The prospect of lowering emissions is one factor leading to the United States’ mandate to produce 136 billion liters of renewable fuel by 2022 [10]. However, meeting this mandate will require substantial land area [11], [12], which implies potentially major changes to regional biogeochemical cycling [12], [13].

Corn grain (*Zea mays*) is the dominant crop used for ethanol production in the US [14]. However, the ability of corn ethanol to reduce GHG emissions is questionable [15], [16], and corn production exacerbates nitrogen pollution and other environmental problems [17]–[19]. Of particular concern is the possibility that diversion of corn for ethanol production will increase global grain prices and trigger agricultural expansion and deforestation elsewhere in the world [7]. The emerging commercial technology to convert ligno-cellulose to ethanol could redress the reliance on corn grain as an ethanol feedstock [20]. This could be particularly beneficial if cellulosic biofuel crops are grown on land that is not important for food production, while having lower GHG emissions than traditional row-crop agriculture [21], [22]. Therefore, considerable research has focused on understanding the soil C and greenhouse gas consequences of replacing traditional agriculture used for bioenergy with perennial grasses like switchgrass (*Panicum virgatum* L.), Miscanthus (*Miscanthus x giganteus* J. M. Greef & Deuter ex Hodk. & Renvoize), or restored prairie cropping systems in the Midwestern United States [17], [23]–[27].

The Southeastern United States holds particular potential for cultivation of second-generation biofuel crops [28], [29]. In comparison with the corn-soy and wheat belts of the Midwestern US, this region’s longer growing season, high precipitation and relatively lower land costs make it attractive for biofuel crop production. However, far less is known about the biogeochemical consequences of land-use change to biofuel crop production in this region.

Energy cane, a promising crop for ligno-cellulosic fuel production, is a variety of sugarcane (*Saccharum officinarum*) that is higher yielding, more cold tolerant and has lower sucrose content than commercially produced sugarcane [28]. Because of its lower sugar concentration, it has not been widely cultivated, but has been of interest commercially as a genetic stock for improving cold tolerance in higher sucrose sugarcane strains [28]. With the development of ligno-cellulosic ethanol conversion technologies, sucrose concentration is less important for ethanol production, and energy cane could become an important biofuel feedstock as yields are high, ranging from 25–74 Mg⋅ha^−1^⋅ yr^−1^ dry mass (Table 1).

**Table 1:** Input parameters (mean and one standard error of the mean; SEM) for carbon and nitrogen concentration of energy cane and soils collected from the Highlands Ethanol farm, Highlands County, Florida.

		*%C*		*%N*	
		Mean	SEM	Mean	SEM
Energy cane	Live leaves	43.68	0.22	1.80	0.18
	Dead leaves	39.77	0.22	0.52	0.03
	Stalks	41.18	0.33	0.87	0.10
Soils	Soil Depth				
Histosols	0–60 cm	7.77	2.48	0.50	0.20
	60–100 cm	7.77	2.48	0.50	0.20
Spodosols	0–30 cm	0.77	0.17	0.04	<0.01
	30–60 cm	0.36	0.03	0.02	0.01
	60–100 cm	0.36	0.03	0.02	0.01

When site-specific data were not available, plant information was used from reference [65], and soil data were collected from the NRCS Web Soil Survey (http://websoilsurvey.nrcs.usda.gov/).

Florida is the largest sugarcane producing state in the US and is therefore a likely location for large-scale energy cane production [30]. Currently, 466,000 hectares of land in Florida are used for low-intensity grazing, and converting some portion of this land could provide an option for growing energy cane [31], [32]. However, it is unknown if converting pasture to cultivated land will affect GHG exchange with the atmosphere and soil carbon storage. More frequent soil disturbance and the presence of larger quantities of litter from growing energy cane could increase CO_2_ efflux to the atmosphere [33], [34], while removing cattle from the landscape will displace methane (CH_4_) efflux [35]. If fields are fertilized, nitrous oxide (N_2_O) emissions may increase because of greater substrate availability for denitrifying microbes [36], and indeed, high rates of N_2_O efflux have been measured from sugarcane grown on highly fertilized soils in Australia [37]. However, considering the entire suite of greenhouse gasses, there may be an overall reduction in GHG flux due to the offset provided by greater atmospheric carbon uptake into the crop.

The region of Florida where energy cane is likely to be grown has two distinct soil types. The most common soils are Spodosols, which are low nutrient and low organic matter sands requiring significant fertilizer to maintain agricultural productivity [38]. Substantial sugarcane production in Florida also occurs on Histosols, which are high organic matter “mucks” that are not typically fertilized, as production on these soils can be maintained by N mineralization from organic matter [39]. The cultivation of Histosols began by draining swamplands, where organic matter had accumulated under anaerobic conditions. Drainage accelerates decomposition and further cultivation of these organic soils is associated with rapid oxidation of organic matter, resulting in significant soil C loss and emissions of CO_2_ and N_2_O to the atmosphere [9], [40], [41].

Theoretical [13], [25] and empirical research [42], [43] indicate that the conversion of land in the rain-fed Midwest currently used to produce corn for ethanol to perennial biofuel feedstocks such as switchgrass or Miscanthus (a close relative of sugarcane) would greatly reduce or reverse the emission of GHG to the atmosphere and rebuild depleted carbon stocks in the soil. Prior studies with Miscanthus in Europe have measured substantial decreases in nitrogen use, and large increases in soil biomass and organic matter relative to other agricultural land uses [44], [45]. There have been no experimental studies that address how changing a landscape to cultivate energy cane will impact GHG emissions and soil C stocks. This is addressed here by using the process-based biogeochemical model DayCent to run *in silico* experiments to ask how land use change from pasture to energy cane production changes ecosystem GHG flux and soil C storage. We test the hypotheses that converting pastures to energy cane will lead to reductions in GHG flux to the atmosphere and increase soil C stocks, and that soil type is an important modulator of that change.

## Methods

### Plant and Soil Analyses

To parameterize the DayCent model, plants and soils were collected on private land in Highlands County, Florida (27° 21′ 49″ N, 81° 14′ 56″ W) in May 2011. Paired 4-m^2^ plots (*n*  = 3) were randomly located in energy cane fields that had been recently (<2 months) converted from pasture and in adjacent non-cultivated pasture on both Spodosols (hyperthermic Arenic Alaquods) and Histosols (hyperthermic Histic Glossaqualfs). We harvested all aboveground biomass from each plot. Soil samples were taken from the pastures in areas not yet under energy cane cultivation. Three soil cores to a depth of 1 m were extracted from each plot with a 1.75-cm diameter wet sampling tube (JMC product # PN010, Newton, IA). Soil cores were separated by depth (0–30 cm, >30 cm). Plant material and soils were oven dried at 65°C (plant material) and 105°C (soils) until they reached constant mass. Dried soils were coarse ground with a mill (model F-4, Quaker City, Phoenixville, PA), and then fine ground with a coffee grinder (Sunbeam Products Inc., Boca Raton, FL). Total C and N content may have been slightly underestimated from the dried Histosols due to volatilization, but the values we measured (Table 1) fall well within the range reported by NRCS Web Soil Survey [46]. Plant material was ground to pass a 425-µm mesh (Wiley mill, Thomas Scientific, Swedesboro, NJ, USA). Plant and soil subsamples within each plot were combined, and C and N concentrations were measured for depth-stratified soil samples (Table 1) and total above ground biomass with a flash combustion chromatographic separation elemental analyzer (Costech 4010 CHNSO Analyzer, Costech Analytical Technologies Inc. Valencia, CA). The instrument was calibrated with acetanilide obtained from Costech Analytical Technologies, Inc. Other physical soil attributes, including texture, bulk density and water holding capacity were obtained from the NRCS Web Soil Survey [46] for Highlands County, Florida.

### The DayCent Model

The DayCent model [47], [48] was developed to simulate ecosystem dynamics for agricultural, forest, grassland and savanna ecosystems [49]–[51]. The model is a daily time step version of the Century model [52], [53], using the same soil carbon and nutrient cycling submodels to simulate soil organic matter dynamics (C and N) and nitrogen mineralization. DayCent uses more mechanistic submodels than Century to simulate daily plant production, plant nutrient uptake, trace gas fluxes (N_2_O, CH_4_), NO_3_ leaching, and soil water and temperature [48], [54]–[58].

The DayCent soil organic matter model is widely used to simulate the impacts of management practices on soil carbon dynamics and nutrient cycling. Specifically, the soil organic matter submodel has been used to simulate the impacts of soil tillage practices; no-tillage, minimum tillage and conventional tillage [59], [60], crop rotations [59], and biofuel crops; woody biomass, switchgrass (*Panicum virgatum*), Miscanthus (*Miscanthus* X *giganteus*), and sugarcane [13], [61] on soil carbon dynamics for agricultural systems. These studies test model performance against observed data and demonstrate general success in simulating changes in soil carbon levels associated with management practices.

The soil trace gas submodel has been extensively tested using observed soil CH_4_ and N_2_O data sets from agricultural and natural ecosystems, and once parameterized with plant production data, provides accurate predictions of trace gas fluxes. Specifically, DayCent has successfully simulated the observed impacts of N fertilizer additions and cropping systems [50], [58], [59] on soil N_2_O and CH_4_ fluxes. The model results and observed data sets demonstrate that increasing N fertilizer levels increases soil N_2_O fluxes and that soil N_2_O fluxes are much lower for perennial crops as compared to annual crops.

The DayCent model has been used extensively to simulate grassland and crop yields [50], [58], [59], [62], and to evaluate the environmental impacts of growing crops. Adler et al. [63] used the DayCent model to simulate net greenhouse gas fluxes (soil C status and soil CH_4_ and N_2_O fluxes) associated with the use of corn, soybeans, alfalfa, hybrid popular, reed canary grass and switchgrass for biofuel energy production in Pennsylvania. Davis et al. [25] used the DayCent model to simulate the environmental impacts of growing switchgrass and Miscanthus in Illinois and compared simulated plant production for switchgrass and Miscanthus with observed yield data. The authors also compared the net soil greenhouse gas fluxes (soil C changes and soil CH_4_ and N_2_O fluxes) associated with growing switchgrass and Miscanthus and growing corn and soybeans. Davis et al. [13] recently used the DayCent model to simulate the environmental impact of replacing the corn currently grown for ethanol production in the Corn Belt with perennial grasses (Miscanthus and switchgrass) for second-generation biofuel production. The authors found that the DayCent model successfully predicted corn, Miscanthus, and switchgrass biomass production for U.S. sites with multiple N fertilizer levels. They also showed that the DayCent model successfully simulated observed annual soil N_2_O fluxes from corn and switchgrass grown with multiple N fertilizer levels and showed that soil N_2_O fluxes are much lower for fertilized switchgrass than for corn.

Furthermore, the basis for the DayCent model, Century, has been used to simulate sugarcane production in Brazil [61], [64] and Australia [65]. These authors show that the Century/DayCent soil organic matter sub-model can correctly simulate the impacts of fertilizer, and organic matter additions on soil carbon levels and surface litter decay.

### Model Parameterization

Energy cane is a variety of sugarcane, and thus parameterizing DayCent for this crop required only minor changes to the previously published input data used for sugarcane [61], [65]. Energy cane differs from sugarcane in that it has increased cold tolerance, decreased sucrose content, and higher cellulose content. We adjusted parameters based on direct measurement of energy cane tissue traits described above. The principal changes from sugarcane to energy cane were reducing the minimum C:N ratio of leaves from 28.6 to 22.1 and changing the C:N of stems from 160 to 30.5. Because of this change in C:N, the parameter for C allocation to stems in DayCent also was modified (from 60% to 40%), to reflect the lower C content of stems relative to N for energy cane versus the previously modeled sugarcane parameters [65]. Bahiagrass (*Paspalum notatum* Flueggé) pasture was simulated using the existing DayCent model parameters for warm season grasses [66], [67].

Histosols are challenging to model as organic matter and C content typically are uniform throughout the soil profile [68], but they also are known to subside because of oxidation of the highly labile organic C pools characteristic of these soils [69]. This subsidence was calculated from the modeled rate of organic matter loss and bulk density. DayCent simulates soil C flux to a depth of 30 cm [47], so as soil was lost with subsidence new soil and organic matter became part of this upper 30 cm column from below. This assumes that loss only occurred in the upper 30 cm, which is reasonable since this is the disturbed and aerated part of the soil. The C and N added from low in the soil profile was calculated from the rate of subsidence and the measured elemental contents and bulk density of the soil that was below 30 cm, when sampled, which is at time zero in our model. However, model output calculates GHG and soil C to soil depths to 30 cm.

### Model Validation

Literature values of aboveground production (dry mass) for grazed pasture, sugarcane and energy cane (Table 2) were used to validate DayCent. Validation focused on aboveground biomass production because this variable has been measured widely across a range of sites. While there were insufficient data on trace gas flux or changes in soil C in sugarcane or energy cane for validation of these variables, validation based on productivity for other crops reliably predicts trace gas flux [59], [60], [63], [70]–[72].

**Table 2:** Site information for studies used in DAYCENT model validation.

*Site*	*Lit. Yield*	*Model Yield*	*Max. Temp.*	*Min. Temp.*	*Precipitation*	*Latitude*	*Longitude*	*Reference*
Auburn, AL	26.1	25.4	24.2	9.8	1160	32.67	−85.44	Woodard and Prine, 1993
Belle Glade, FL	25.0	28.3	27.8	16.4	1378	26.68	−80.67	Korndorfer, 2009
EREC, FL	51.3	43.5	29.1	17.7	1181	26.65	−80.63	Gilbert et al., 2006
Gainesville, FL	35.6	27.3	27.0	13.7	1123	29.68	−82.27	Woodard and Prine, 1993
Hendry, FL	39.2	55.9	28.4	18.3	1362	27.78	−82.15	USDA, 2011
Hillsboro, FL	60.7	62.5	28.5	18.3	1547	27.90	−82.49	Gilbert et al., 2006
Houma, LA (1st ratoon)	36.6	38.2	25.2	14.8	500	29.57	−90.65	Legendre and Burner, 1995
Houma, LA (2nd ratoon)	34.9	37.6	25.2	14.8	500	29.57	−90.65	Legendre and Burner, 1995
Hundley, FL	73.5	62.0	28.5	18.1	1457	26.30	−80.16	Gilbert et al., 2006
Jay, FL (plant cane)	35.8	33.6	26.9	16.4	1321	28.65	−80.82	Woodard and Prine, 1993
Jay, FL (1st ratoon)	27.8	32.8	26.9	16.4	1321	28.65	−80.82	Woodard and Prine, 1993
Lakeview, FL	71.3	62.5	28.5	17.4	1275	26.30	−80.15	Gilbert et al., 2006
Hidalgo, TX	34.6	42.5	28.7	18.1	576	26.17	−97.93	Weidenfeld, 1995
Ona, FL (1st ratoon)	40.5	38.1	28.6	16.0	1160	27.48	−81.92	Woodard and Prine, 1993
Ona, FL (2nd ratoon)	30.2	31.6	28.6	16.0	1160	27.48	−81.92	Woodard and Prine, 1993
Pahokee, FL	60.5	65.5	28.4	17.5	1269	26.82	−80.66	Glaz and Ulloa, 1993
Palm Beach, FL	32.3	35.4	29.0	16.6	851	26.67	−80.15	USDA, 2011
Quincy, FL (1st ratoon)	26.3	25.1	25.8	12.9	1445	30.59	−84.58	Woodard and Prine, 1993
Quincy, FL (2nd ratoon)	27.8	21.7	25.8	12.9	1445	30.59	−84.58	Woodard and Prine, 1993
Shorter, AL	26.4	25.4	24.9	10.9	1119	32.40	−85.94	Sladden et al., 1991
Sundance, FL	42.1	43.5	28.6	17.5	1303	26.60	−80.87	Gilbert et al., 2006

Yield values from the literature and modeled yields for energy cane and sugarcane represent total aboveground biomass expressed as Mg ha^−1^ on a dry mass basis. Climate variables include mean annual maximum and minimum temperature (°C) and mean annual precipitation (mm).

We compiled a literature database of 17 sites that had reliable data on sugarcane and energy cane yield. There were also pasture productivity data for 15 of those sites [73]. In some instances we were able to contact researchers directly to access unpublished data (Table 2). The geographic range of sites represents the breadth of sugarcane production in the continental United States, and the potential range of energy cane production on currently grazed pastures. For all sites, daily weather data inputs (minimum and maximum temperature, daily precipitation) from 1980 to 2002 were obtained from the DayMet database [74]. The model was run using the DayCent growing degree-day subroutine to determine plant emergence, senescence and death, based on plant phenological characters and daily weather data. Soil data for the validation sites were obtained from the NRCS Web Soil Survey [75]. Using the same schedule of management events used for the *in silico* experiments (described below), DayCent was run with site-specific soil and weather data for each sites. The fit of modeled to measured above ground dry mass production (Mg dry matter^.^ ha^−1^) of our simulations of grazed pasture and energy cane were separately tested via linear regression, using the linear model function in R [76].

### Initial Simulation Conditions

A “spin-up” period in DayCent based on historical land use and vegetation type was used to set initial soil conditions. The dominant, historic vegetation type for this area of south-central Florida was savanna, with a mixture of grasses and several species of scrub-oak, or sawgrass for the swamp areas [77]. A mix of perennial C_3_ grasses species and symbiotic N_2_ fixing plants, were used as initial conditions for the savanna simulation (initial vegetation type “savanna” in DayCent). A period of 2000 years was simulated to obtain an initial soil C and N conditions prior to our *in silico* experiments. The model was run for spin ups and all subsequent experiments using the growing degree-day sub-routine.

### In silico Experiments

Model simulations were then run to determine the GHG soil-atmosphere exchange and change in soil C predicted for conversion of pasture to energy cane on the two dominant soil types, Spodosols and Histosols. We used daily weather data inputs (minimum and maximum temperature, daily precipitation) from 1951 to 2002, which was the longest time period available for Highlands County, Florida obtained from the DayMet database [74]. This weather file is used by DayCent to create a mean and standard deviation of weather parameters, thus the more weather data available for a given site, the more accurately the variability of a site will be captured by the model.

To initiate the experimental simulations, in 1998 we converted the savanna by removing all above ground biomass and plowing to a depth of 30 cm. A landscape conversion to a grazed Bahiagrass (*Paspalum notatum* Flueggé) ecosystem was then simulated. Bahiagrass is a common forage grass for this part of Florida that would be considered “improved pasture”, although usually not fertilized or irrigated [78]. We simulated grazing in our modeling experiment by annually removing 10% of live shoot and 1.0% of standing dead shoots. Prior to planting energy cane, another plow event to 30 cm was initiated to remove the pasture vegetation and simulate the physical land use change.

The simulated cycle of energy cane planting and harvest was based on the sugarcane literature [65], [79], [80] and discussions with University of Florida and USDA sugarcane agronomists [81], [82]. In the simulations, energy cane was planted in January of the first year (2013), followed by a two-year ratoon (crop regenerated from remaining biomass) from which 80% of the above ground biomass was harvested in December. At the end of the second ratoon, the crop was removed and the land plowed before planting a new plant crop. This cycle of ratooning and planting was repeated in the simulation for fifteen years following conversion from pasture; i.e. five cycles of three years each. This three-year planting cycle is typical for sugarcane production in Florida [83], [84].

Irrigation events were scheduled every month throughout the dry season, and every two months during the rainy season to maintain soil water at field capacity. Fertilizer (NH_4_^+^ – NO_3_^−^) was applied in mid February and mid June of each year of the simulation, at a rate of 102 kg N^.^ ha^−1^ per fertilization event for Spodosols. No fertilizer was added to the organic rich Histosols. This fertilization schedule was based on studies that suggest that a split fertilization regime at this rate maximizes sugarcane yield, and that fertilizing above this level does not increase yield but increases N_2_O efflux [38], [65], [85]. The input files used to drive DayCent (e.g. schedule files, plant input parameters, and soil input files) are available online [86].

### Calculations and Statistical Analyses

We summed daily GHG and soil C fluxes from DayCent to calculate yearly fluxes and report those in g C or N⋅m^−2^ yr^−1^, with the exception of total GHG values which are reported as CO_2_ equivalents [87] and factored by warming potential (CO_2_ = 1, CH_4_ = 23, N_2_O = 296; ref. 85). Total ecosystem C flux was calculated as the annual change in total ecosystem C storage between the beginning and end of a year and represents the net ecosystem carbon balance expressed in CO_2_eq [88], [89].

Because the model experiments were performed using the same site with the same weather data, but controlled for soil type, the simulations had the structure of a paired design where each year was a replicate [90]. We therefore used paired t-tests to determine differences between soil types within a plant type (*n*  = 15) and between plant types within a soil type (*n*  = 15). The variation reported with mean annual values represents inter-annual variation in the predicted variables. Heteroscedasticity was examined with the Fligner-Killeen test, and output data distributions, which did not meet variance assumptions, were compared with the Wilcoxon rank-sum test. The routines t.test (paired  =  TRUE) and wilcox.test were performed using R [76], [90].

Because of the large number of pair-wise comparisons of our model results, the False Discovery Rate (FDR) test was used to account for multiple comparisons. The FDR test is less conservative than a *P*-value adjustment such as the Bonferroni correction, and determines the probability of a Type I error. We calculated a FDR of 0.024 for our matrix of tests, and therefore justified the use of multiple paired t-tests without *P*-value adjustment [91].

## Results

**Figure 1:**
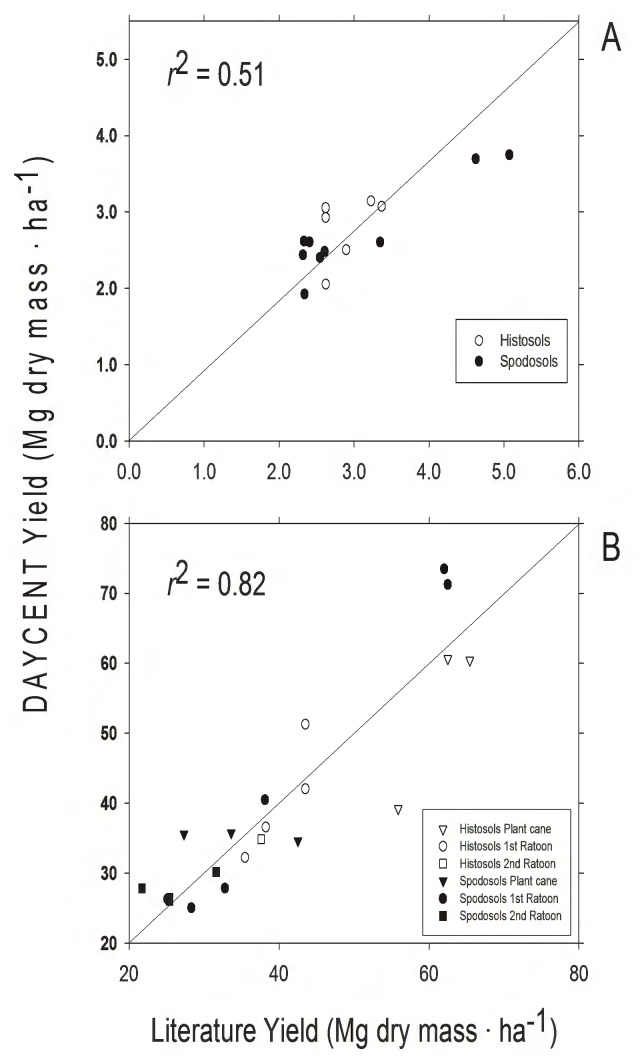
Regression analysis used in DayCent model validation. Model output for dry mass yield was compared to literature values for A) pasture yield values from the USDA-NASS database, B) sugarcane and energy cane dry mass yield. Data points are compared to a 1∶1 line.

Predicted harvested yields for both pasture and energy cane in our validation sites agreed well with measured values from the literature (Pasture: *r* ^2^ = 0.52, Energy cane: *r* ^2^ = 0.82, Figure 1A & 1B), indicating that our modeled predictions provided a good representation of the productivity that drives the biogeochemical dynamics of DayCent.

**Figure 2:**
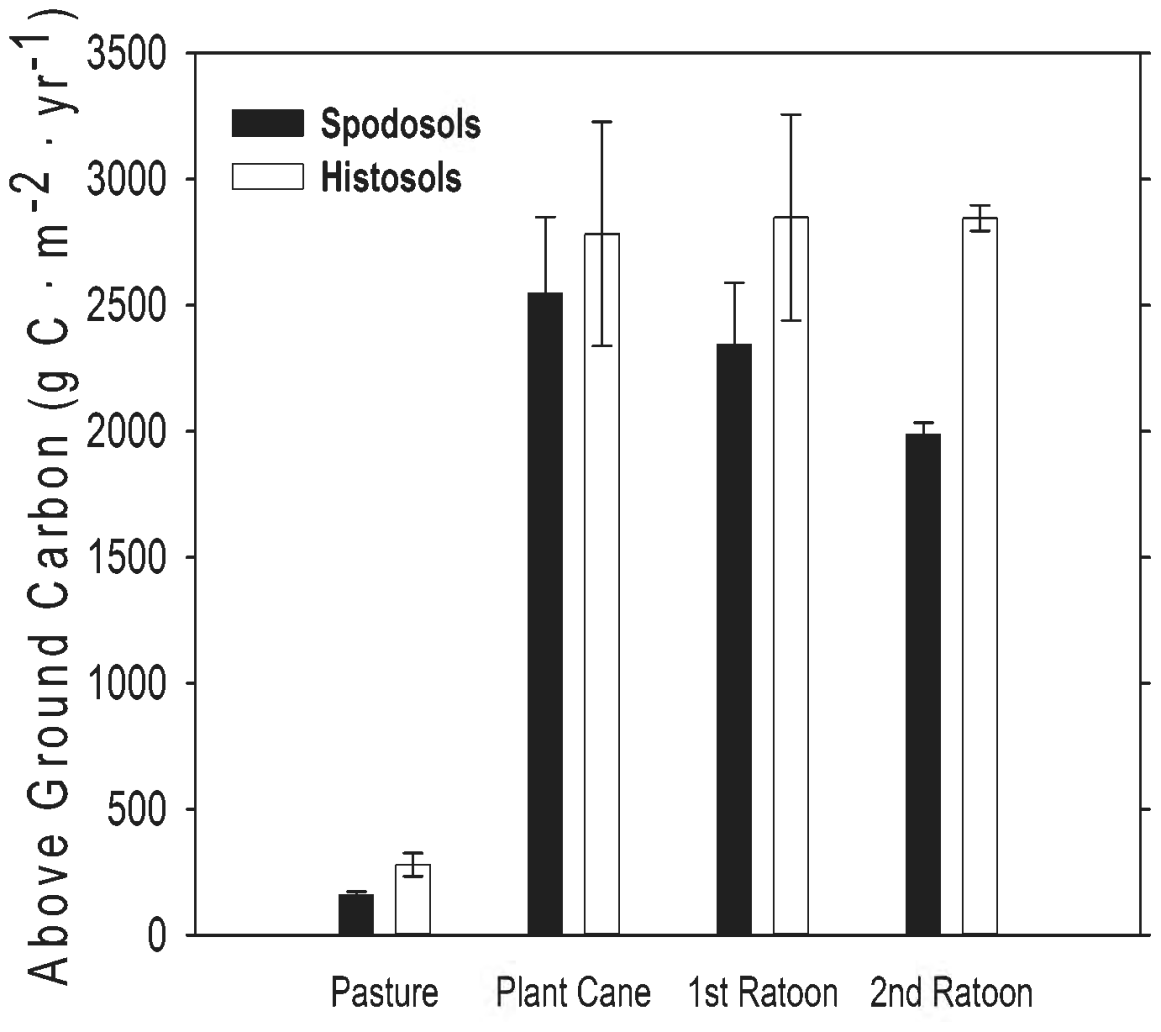
Modeled above ground production of grazed pasture and energy cane in Highlands County, Florida. Values are mean above ground carbon (g C⋅m^−2^⋅yr^−1^, ± SD) for 15 years in pasture, and for 5 × 3-year ratoon cycles in energy cane (each bar represents the average of 5 values, one for each year for each stage in the planting cycle).

For our modeled site, DayCent estimated a large increase in aboveground plant biomass production after conversion of pasture to energy cane (Figure 2); annual aboveground biomass production increased by a factor of 14 on Spodosols and by a factor of 10 on Histosols, relative to pasture. Energy cane production ranged from 1911–3153 g C m^−2^ yr^−1^ (46–76 Mg dry biomass⋅ha^−1^). Predicted energy cane production remained high through the three harvests on Histosols, but declined through the modeled ratoon cycle on Spodosols (Figure 2).

**Figure 3:**
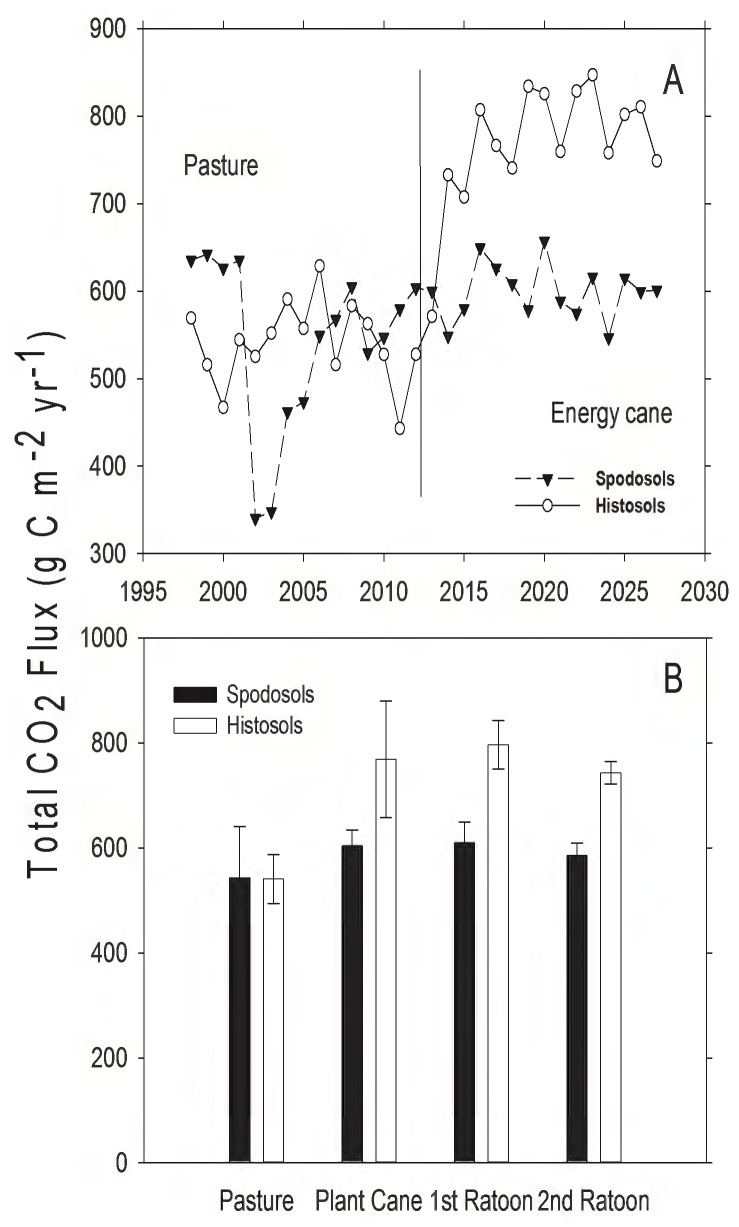
Modeled total soil CO_2_ flux from pasture and land converted to energy cane in Highlands County, Florida. A) Total annual soil CO_2_ flux (expressed as g C⋅m^−2^). Dashed line represents year of land use conversion from pasture to energy cane. B) Mean total soil CO_2_ flux (g C⋅m^−2^⋅yr^−1^, ± SD) for 15 years in pasture, and for 5, 3-year ratoon cycles in energy cane (each bar represents the average of 5 values, one for each year for each stage in the planting cycle).

There was considerable temporal variation in predicted soil CO_2_ efflux from pasture in the 15 years simulated prior to the conversion to energy cane (Figure 3a). This variation was particularly evident for pasture on Spodosols and was driven primarily by variation in precipitation. Total soil CO_2_ efflux was similar for pasture on both soil types, but significantly increased when averaged over 15 years after conversion to energy cane on the Histosols (Figure 3a; *t*  = 10.65, d.f.  = 14, *P*<0.001). Land use conversion did not increase CO_2_ efflux on Spodosols (*t*  = 0.58, d.f.  = 14, *P*  = 0.57). Following conversion to energy cane CO_2_ efflux from Histosols was significantly higher than energy cane on Spodosols (Figure 3b; *t*  = 9.56, d.f.  = 14, *P*<0.001).

**Figure 4:**
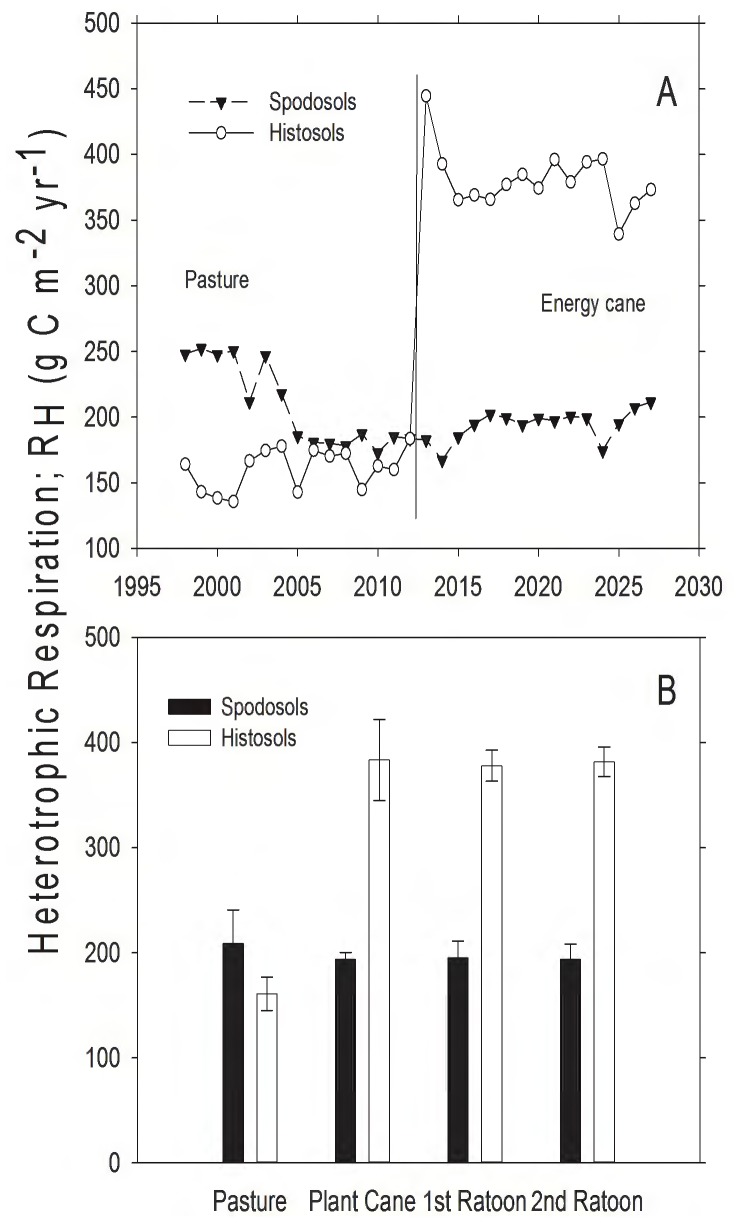
Modeled heterotrophic respiration (R_H_) from pasture and land converted to energy cane in Highlands County, Florida. A) Total annual heterotrophic respiration (g C⋅m^−2^). Dashed line represents year of land use conversion from pasture to energy cane. B) Mean heterotrophic respiration (g C⋅m^−2^⋅yr^−1^, ± SD) for 15 years in pasture, and for 5, 3-year ratoon cycles in energy cane (each bar represents the average of 5 values, one for each year for each stage in the planting cycle).

The conversion of land from pasture to energy cane had no significant effect on the predicted heterotrophic component of soil respiration (R_H_) on Spodosols (Figure 4a), but caused a large increase in R_H_ from the Histosols (Figure 4a; *t*  = 31.86, d.f.  = 14, *P*<0.001) and resulted in higher R_H_ on Histosols than Spodosols following the conversion to energy cane (Figure 4b; *t*  = 23.68, d.f.  = 14, *P*<0.001). Prior to the conversion to energy cane, modeled (R_H_) was slightly higher in pasture on Spododols than on Histosols (Figure 4; *t*  = 31.86, d.f.  = 14, *P*<0.001).

**Figure 5:**
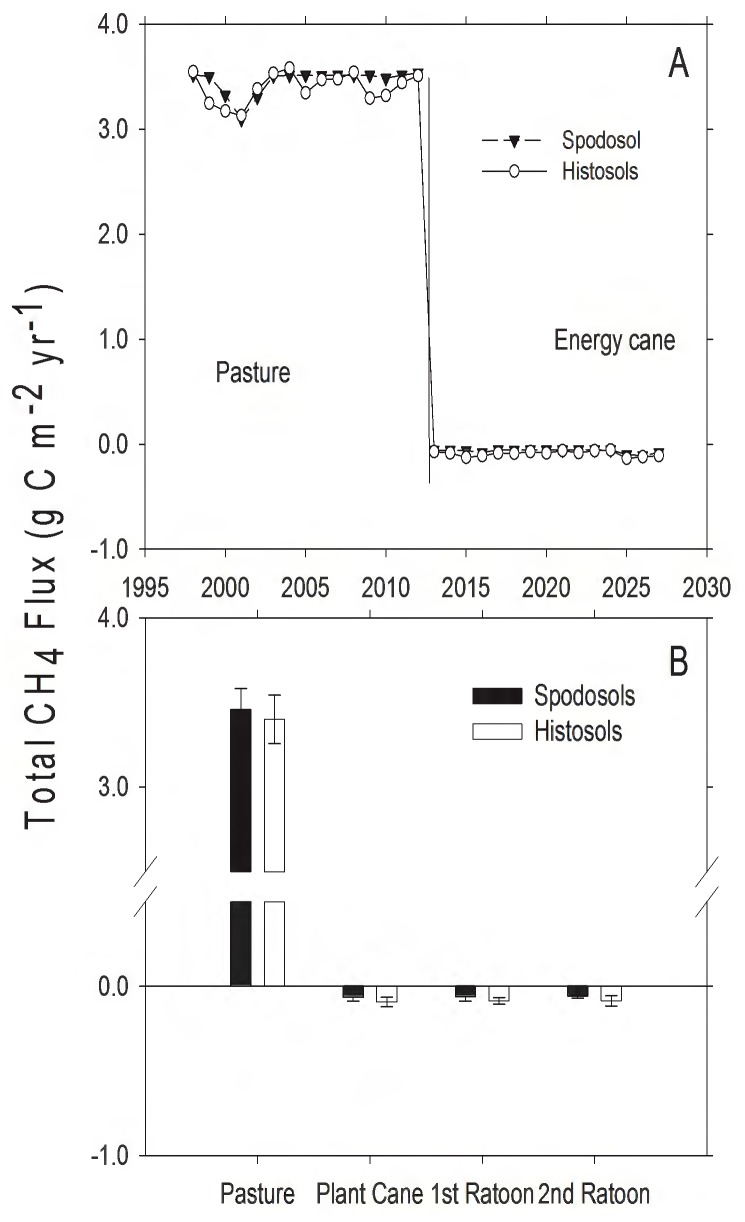
Modeled CH_4_ flux from pasture and land converted to energy cane in Highlands County, Florida. A) Total annual CH_4_ flux (g C⋅m^−2^). The solid vertical line represents year of land use conversion from pasture to energy cane, positive values indicate CH_4_ efflux and negative values indicate CH_4_ uptake. B) Mean CH_4_ flux (g C⋅m^−2^⋅yr^−1^, ± SD) for 15 years in pasture, and for 5, 3-year ratoon cycles in energy cane (each bar represents the average of 5 values, one for each year for each stage in the planting cycle).

On both soil types, the removal of cattle associated with the conversion of pasture to energy cane caused a substantial change in predicted CH_4_ flux (*t*  = 185, d.f.  = 14, *P*<0.001 on Spodosols; *t*  = 167, d.f.  = 14, *P*<0.001 on Histosols; Figure 5a). Without cattle, pastures were a small CH_4_ sink (0.16–0.60 g C⋅m^−2^⋅yr^−1^ uptake in Spodosols, 15 year sum  = 112 g CO_2_eq⋅m^−2^, 0.12–0.57 gC⋅m^−2^⋅yr^−1^ uptake for Histosols, 15 year sum  = 135 g CO_2_eq⋅m^−2^). Introducing cattle at stocking rates and grazing intensity typical for this region (1 head cattle⋅ha^−1^: ref. 31), caused pasture on both soil types to be a substantial source of CH_4_ to the atmosphere (Figure 5).

**Figure 6:**
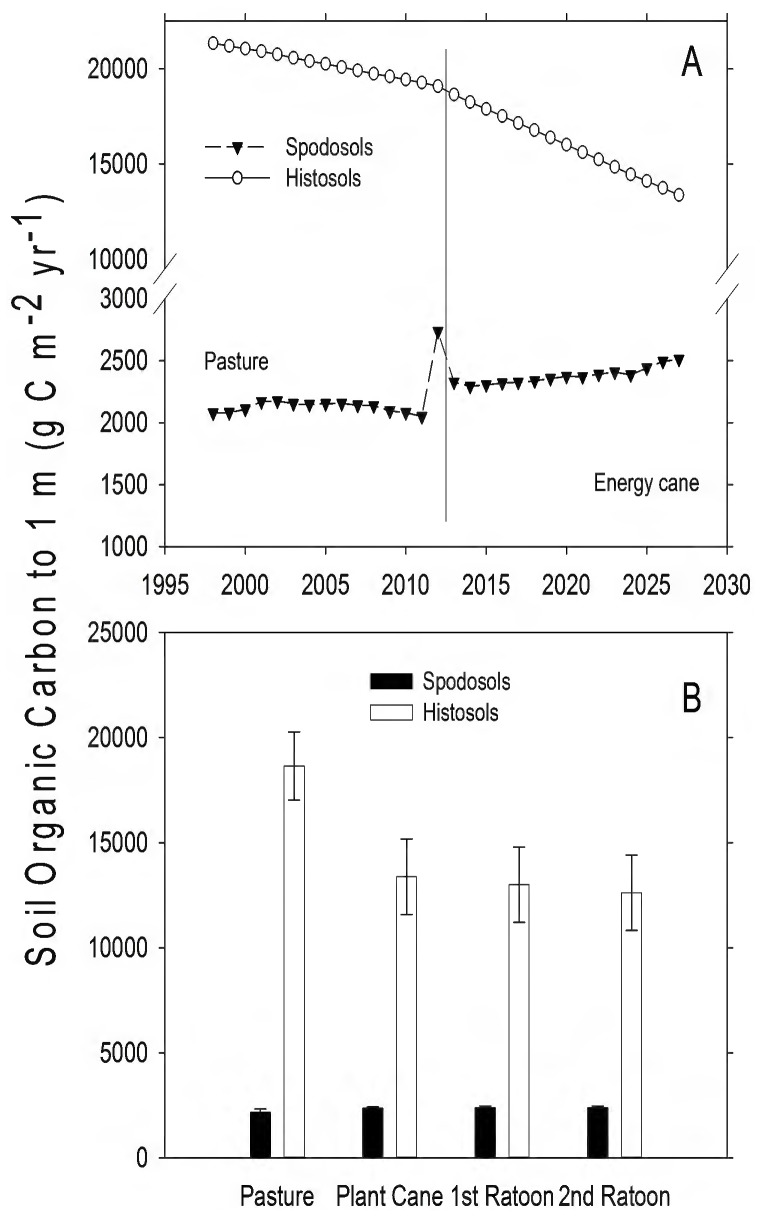
Changes in total soil organic C from pasture and land converted to energy cane in Highlands County, Florida. A) Total annual SOC flux (g C⋅m^−2^). The solid vertical line represents year of land use conversion from pasture to energy cane. B) Mean SOC flux (g C⋅m^−2^⋅yr^−1^, ± SEM) for 15 years in pasture, and for 5, 3-year ratoon cycles in energy cane (each bar represents the average of 5 values, one for each year for each stage in the planting cycle).

Changes in vegetation and management practices altered soil organic carbon (SOC), and these changes were particularly evident on the Histosols (Table 2; Figure 6). Histosols had a larger pool of active C (weekly to monthly turnover) than Spodosols under both pasture and energy cane (pasture, *t*  = 19.25, d.f.  = 14, *P*<0.001; energy cane, *t*  = 14.21, d.f.  = 14, *P*<0.001). Comparing the remaining total SOC pools between the end of pasture and the last year of the energy cane simulation, Histosols lost a large amount of soil organic C; 5714 g C⋅m^−2^ to 1 m depth (Figure 6; *t*  = 296, d.f.  = 14, *P*<0.001), compared to the SOC loss from Spodosols of 224 g C⋅m^−2^ to 1 m (Table 3).

**Table 3:** Modeled ecosystem carbon, nitrogen and greenhouse gas fluxes after converting pasture to energy cane on nutrient poor Spodosols and organic matter rich Histosols.

		Spodosols			Histosols	
	Pasture	Energy cane	Δ	Pasture	Energy cane	Δ
SOC (g C⋅m^−2^)	2736	2513	−224	16087	10373	−5715
Nitrogen Mineralization (g N⋅m^−2^)	134	203	69	216	293	77
Heterotrophic Respiration (g C⋅m^−2^)	3130	2913	−218	2413	5715	3302
Total Soil CO_2_ Efflux (g C⋅m^−2^ )	8148	8993	845	8111	11540	3429
CH_4_ (g CO_2_eq⋅m^−2^)	2980	−33	−3013	2958	−46	−3004
N_2_O (g CO_2_eq⋅m^−2^)	214	649	435	6713	1742	−4970
Total System C Flux (g CO_2_eq⋅m^−2^)	−1159	−2812	−1653	−1367	924	2291
Total Greenhouse Gas Flux (g CO_2_eq⋅m^−2^ )	2035	−2196	−4231	8304	2620	−5684

Greenhouse gas and N mineralization values are the sum of values from pasture 15 years prior to conversion to energy cane and the sum values for 15 years following the conversion to energy cane. Positive values indicate a flux to the atmosphere and negative values indicate uptake from the atmosphere by the ecosystem. Soil organic matter values are the differences between the last year of energy cane production and the last year of pasture. Total GHG values are the sums of CH_4_, N_2_O and total system C flux (calculated in DayCent as the difference between all C uptake and storage versus efflux from respiration) expressed as CO_2_e. Differences (Δ) represent the values for energy cane minus pasture.

**Figure 7:**
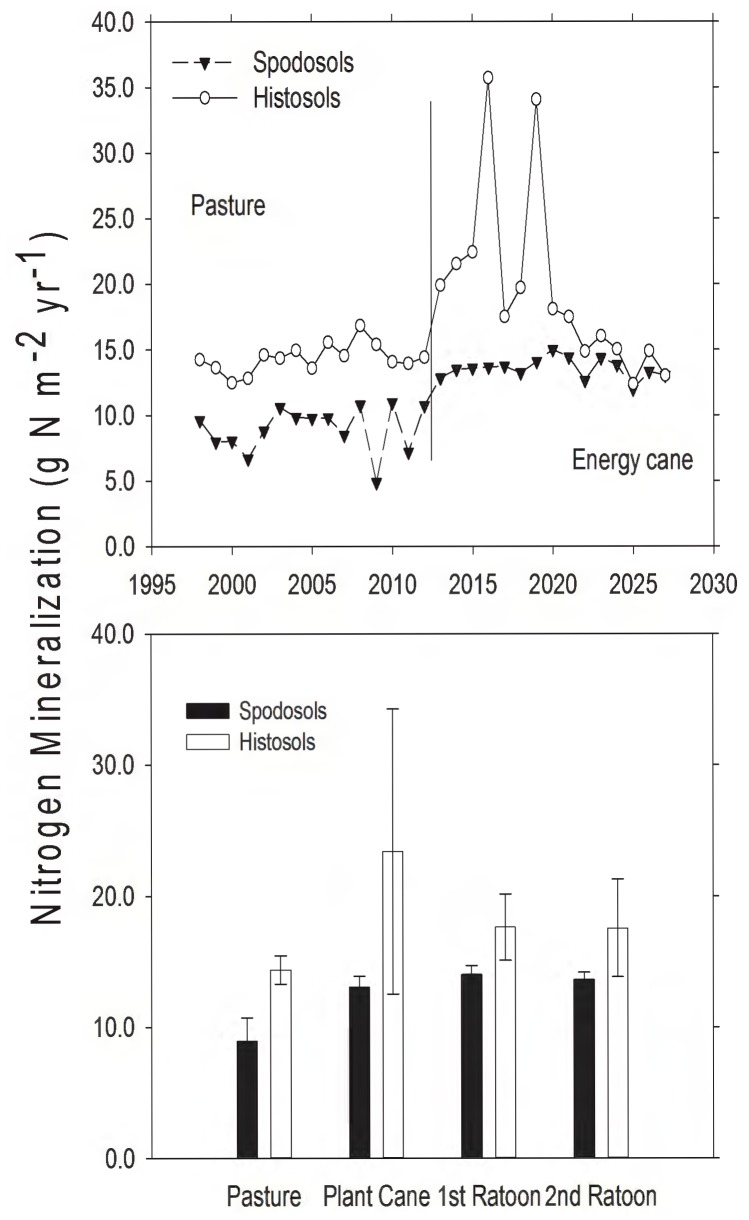
Modeled nitrogen mineralization rates from pasture and land converted to energy cane in Highlands County, Florida. A) Total annual N mineralization rate (g N⋅m^−2^). The solid vertical line represents year of land use conversion from pasture to energy cane. B) Mean N mineralization rate (g N⋅m^−2^⋅yr^−1^, ± SD) for 15 years in pasture, and for 5, 3-year ratoon cycles in energy cane (each bar represents the average of 5 values, one for each year for each stage in the planting cycle).

Nitrogen mineralization increased after pasture was converted to energy cane on both the fertilized Spodosols (*t*  = 9.02, d.f.  = 14, *P*<0.001) and the non-fertilized Histosols (*t*  = 2.72, d.f.  = 14, *P*  = 0.02). After conversion to energy cane, Histosols had higher rates of N mineralization than Spodosols (Figure 7; *t*  = 3.43, d.f.  = 14, *P*  = 0.004), and this increase in available N likely accounted for the continued high yields on Histosols.

**Figure 8:**
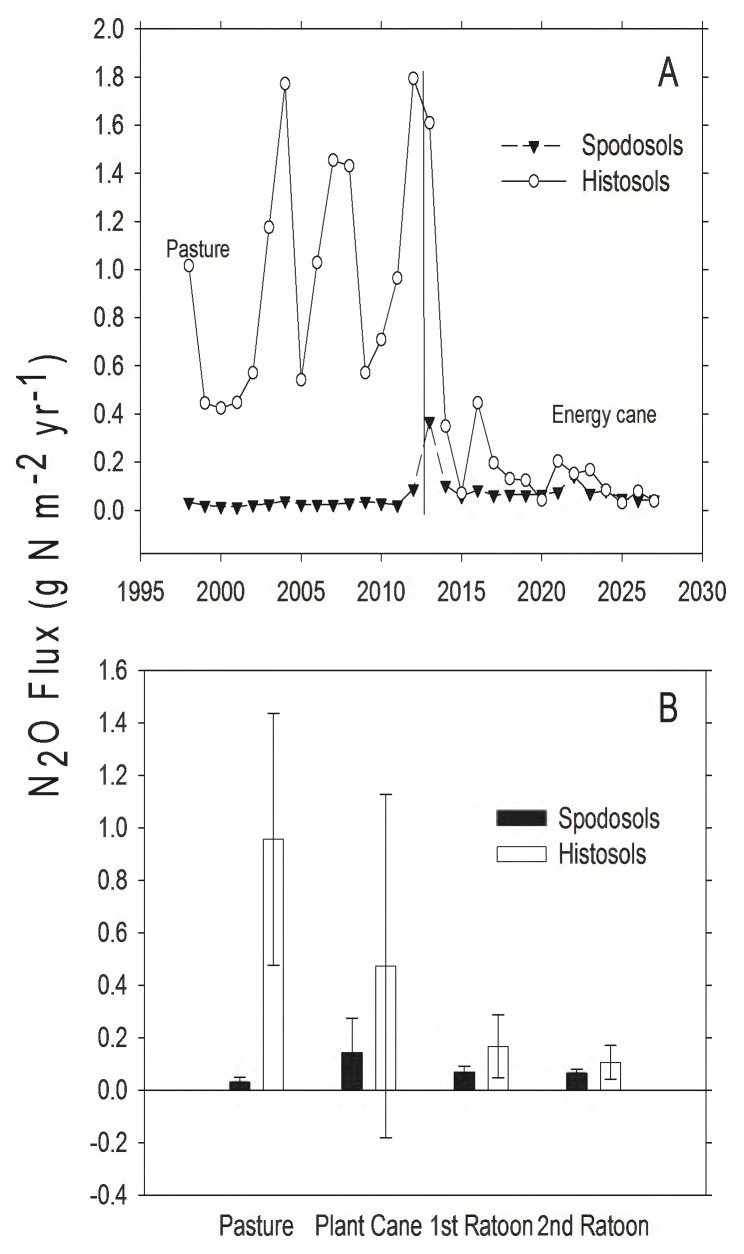
Modeled N_2_O flux from pasture and land converted to energy cane in Highlands County, Florida. A) Total annual N_2_O flux (g N⋅m^−2^). The solid vertical line represents year of land use conversion from pasture to energy cane, positive values indicate N_2_O efflux and negative values indicate N_2_O uptake. B) Mean N_2_O flux (g N⋅m^−2^⋅yr^−1^, ± SD) for 15 years in pasture, and for 5, 3-year ratoon cycles in energy cane (each bar represents the average of 5 values, one for each year for each stage in the planting cycle).

Prior to conversion to energy cane, N_2_O efflux was higher in pastures on Histosols compared to Spodosols (Figure 8; Wilcoxon rank sum, W  = 225, *P*<0.001). After conversion to energy cane, Histosols remained greater sources of N_2_O than Spodosols (*t*  = 12.15, d.f.  = 14, *P*<0.001). Conversion of pasture to energy cane decreased N_2_O efflux on Histosols (Figure 8a; *t*  = 4.30, d.f.  = 14, *P*<0.001), but increased N_2_O efflux on Spodosols (Figure 8b; *t*  = 2.87, d.f.  = 14, *P*  = 0.01). It is likely that N_2_O emission from Histosols decreased following conversion because the increase in productivity resulted in a higher uptake of nitrate that would otherwise be available for denitrification.

**Figure 9:**
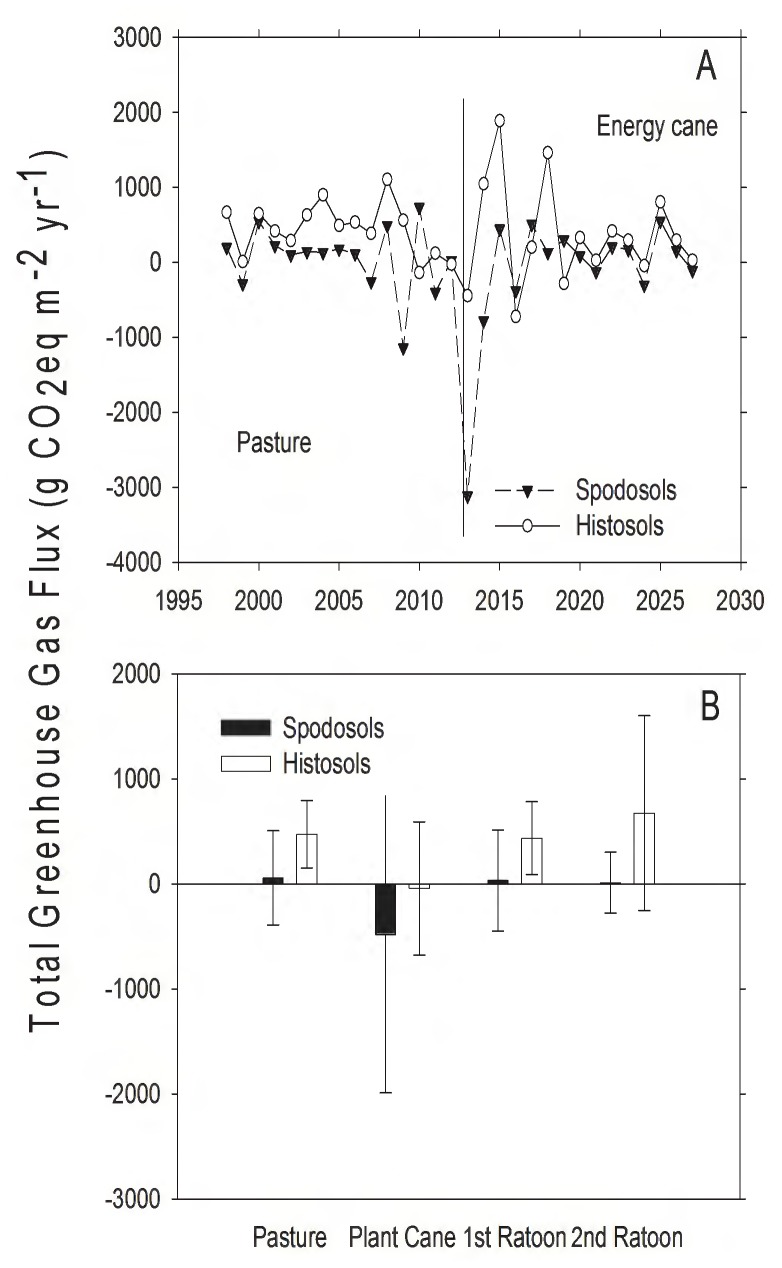
Changes in total greenhouse gas (GHG) from pasture and land converted to energy cane in Highlands County, Florida. Positive values indicate GHG efflux and negative values indicate GHG uptake. A) Total annual GHG flux, reported as CO_2_ equivalents converted to account for differences in warming potential (g CO_2_e⋅m^−2^). The solid vertical line represents year of land use conversion from pasture to energy cane. B) Mean greenhouse gas flux in CO_2_ equivalents converted to account for differences in warming potential (g CO_2_e⋅m^−2^⋅yr^−1^, ± SD) for 15 years in pasture, and for 5, 3-year ratoon cycles in energy cane (each bar represents the average of 5 values, one for each year for each stage in the planting cycle).

Total GHG exchange (global warming potential) was calculated by converting the fluxes of CH_4_ and N_2_O to CO_2_ equivalents based on their warming potential relative to CO_2_ [87] and summing these with total system C flux (Table 2). Variation in weather caused substantial inter-annual variation in total GHG flux, with both pasture and energy cane varying between net GHG sinks and sources (Figure 9); no significant differences in annual GHG flux were resolved on either soil type (Spodosols: *t*  = 1.15, d.f.  = 14, *P*  = 0.27; Histosols: *t*  = 0.13, d.f.  = 14, *P*  = 0.90). When the cumulative GHG emission were calculated for the fifteen years prior to conversion, pasture was a net source to GHGs to the atmosphere on both soil types, and pasture was a stronger source on Histosols (8304 gCO_2_eq⋅m^−2^) than on Spodosols (2035 gCO_2_eq⋅m^2^; Table 3). Conversion of pasture to energy cane caused the Spodosols to transition from a source to a sink for GHGs and reduced the flux of GHGs to the atmosphere on Histosols. On both soil types, the reduction GHG emission to the atmosphere was associated with a large decrease in CH_4_ emissions caused by the elimination of cattle grazing. On the Histosols, the reduction in N_2_O emissions to the atmosphere also contributed to reduced emission of GHGs. This analysis of GHG emissions and their corresponding global warming potentials did not account for the displacement of fossil fuel emissions by the biofuel product.

## Discussion

Parameterization of the DayCent model for energy cane, an emerging bioenergy crop, successfully simulated biomass production across the southeast United States (Figure 1). Our simulations suggested high yields for energy cane on former pastureland in a subtropical climate when Spodosols are highly fertilized (200 kg N⋅ha^−1^⋅yr^−1^), and when microbial activity in Histosols leads to high rates of N mineralization (rates were 44% higher on Histosols). When integrated over 15 years (Table 3), conversion of pasture to energy cane on Spodosols converted a net source of GHG (due to cattle CH_4_ emissions) to a sink driven by the removal of cattle and the increase in C uptake by energy cane. While Histosols were a net GHG source under both pasture and energy cane, the source was reduced by the land use conversion (Table 3). The GHG improvement resulting from this conversion from pasture to energy cane would be even greater if fossil fuel displacement by cellulosic ethanol had been included.

The range of our simulated energy cane yields was 46–76 Mg ha^−1^ dry mass per year on fertilized Spodosols and unfertilized Histosols. Using published values for the conversion efficiency for the production of cellulusoic ethanol [22], a hypothetical energy cane farm of 10,000 ha could therefore produce between 142–236 million liters of ethanol [92]. In comparison, equal areas of land devoted to corn grain and Miscanthus in the Midwest would yield between 25 and 73% this amount of ethanol, respectively, assuming the maximum yields reported by other authors [11], [22].

Typically, sugarcane yield declines with ratooning, the repeated harvests of aboveground material generated by vegetative growth [93]. The model reproduced the yield decline for energy cane on Spodosols but not on Histosols, but the model in its current configuration probably failed to capture the mechanisms that would normally cause a decline in yield. Various factors ranging from increases in nematode populations and ratoon stunting disease, to mechanical compaction of the soil have been implicated in ratoon decline [94], [95], and these were not accounted for in the model. Although sugarcane in Florida typically is grown for three years and three annual harvests before re-planting, if it were grown for more years between re-planting, we would expect a continuing yield decline on the Spodosols. In contrast, continued mineralization of organic matter on Histosols may sustain high yields beyond the 3-year period simulated in the model. On both soils the GHG benefits would be improved with longer ratoon cycles because of less soil disturbance due to decreased frequency of soil disturbance for replanting.

Organic matter (OM) content of soils is important for sustaining high yields of sugarcane, in part because OM mineralization provides the labile N necessary to sustain plant growth. Spodosols had much less OM than Histosols (Appendix I; Figure 6; [65], [96]). The high CO_2_ efflux rates from Histosols (Figures 3–4) and the patterns of SOC loss following land use change (Figure 6) correspond to higher rates of OM mineralization. The associated higher rates of N mineralization (Table 3; Figure 7) on Histosols provided additional N to energy cane and improved crop yield (Figure 2). Although energy cane on Spodosols was fertilized to offset the low N content of these soils, rates of nitrification (the process by which NH_4_^+^ is converted into the highly mobile NO_3_^−^ anion) were higher on these soils. The fertilizer applied to energy cane crops on Spodosols in the simulations was NH_4_^+^ - NO_3_^−^, a labile substrate for nitrification [66]. Spodosols had consistently higher nitrification rates than Histosols, and therefore higher NO_3_^−^ content because of fertilization, and it is possible that some fraction of fertilizer was lost before plant uptake [97]. We hypothesize that a combination of NO_3_^−^ leaching from fertilizer before plant uptake, lower initial N content, and lower mineralization rates may have created a stronger N limitation to yield on Spodosols but not on Histosols.

Before land use change, pasture on both soil types was a net source of GHGs to the atmosphere (Table 3). This is consistent with both direct measurements [98] and modeling efforts [99] that have found grazed pastures to be net sources of GHGs, but this is also a function of grass species present and animal stocking density [100]. The model estimated that pastures were sinks for CO_2_, with total C uptake of 1159 g CO_2_ m^−2^ and 1367 g CO_2_ m^−2^ over 15 years on Spodosols and Histosols, respectively (Table 3). In the absence of cattle, both soil types were CH_4_ sinks (112 and 135 g CO_2_eq, respectively), but including reasonable estimates of CH_4_ efflux from cattle (Figure 5) and N_2_O efflux from soils (Figure 8) resulted in net GHG emission to the atmosphere on both pasture soils (Table 3). Following conversion to energy cane, the production of N_2_O on Spodosols increased (Figure 8) within the range of N_2_O flux rates previously reported for Australian sugarcane fertilized at similar rates to this study [37]. The increase in N_2_O was offset by uptake of CO_2_ and the change from a source to a sink for CH_4_ (Figure 5), with the net effect that Spodosols became a net GHG sink (Table 3). Indeed, over 15 years energy cane on Spodosol was a GHG sink of >40 Mg CO_2_eq per hectare (Table 3). On Histosols, eliminating grazing following the conversion of pasture to energy cane caused a similar decrease in CH_4_ efflux to the atmosphere (Figure 5) and this land use change also reduced N_2_O emissions (Figure 8; Table 3). However, following land conversion this system switched from a net CO_2_ sink to a source, and this change in total system C prevented energy cane on Histosols from becoming a net sink for GHGs. The driver for GHG production on Histosols was higher R_H_, and significant losses of soil organic matter [69] that resulted in total C efflux from these soils (Table 3).

The model successfully simulated energy cane biomass production across a range of sites across the southern United States (Figure 1). Previous studies have shown that DayCent reliably predicts soil biogeochemistry and GHG exchange when parameterized for net primary production [51], suggesting that the estimates of GHG flux and soil C dynamics were reasonable. Eddy-flux measurements of GHG exchange that are now being initiated at this site will provide an independent test of the predictions of GHG effects of conversion made here.

Indirect land use change (ILUC) – the stimulation of deforestation or increased agriculture in other parts of the world driven by diversion of current agricultural land to bioenergy production – potentially poses an environmental risk of bioenergy production [7], [101]. Growing energy cane on land converted from low stocking density pasture would be unlikely to trigger significant increases in food price or ILUC in the way that large-scale shifts from corn or soy production in the Midwestern United States would motivate greater production of those crops elsewhere [13]. Indeed, the recommended stocking density for Bahiagrass pasture in this region is ∼1 animal⋅ha^−1^ [31], and cattle and calf operations in Florida account for less than 6% of the state’s annual agricultural revenue [102]. The loss in meat production could be redressed with minimal increases in current stocking rates, and would be unlikely to trigger the type of large-scale landscape changes that may occur through the diversion of midwestern agricultural land [7]. However, displacing cattle for energy cane production may potentially increase methane emissions elsewhere, which would negate the local benefit of reduced methane flux to the atmosphere.

The environmental impacts of changing land use from pasture to energy cane were highly dependent on the soil type. Whereas the cultivation of Histosols results in high CO_2_ efflux and the reduction of soil carbon (Figures 3, 4, and 6), the model predicted that energy cane crops on Spodosols would act as a net C and GHG sink (Figure 6, Table 3). From both a biofuel and biogeochemical perspective, these results suggest that energy cane grown on nutrient poor soils, as opposed to organic soils, has the potential to be a high-yielding bio-ethanol feedstock that creates a GHG sink in the Southeastern United States.
